# *Staphylococcus aureus* is More Prevalent in Retail Beef Livers than in Pork and other Beef Cuts

**DOI:** 10.3390/pathogens4020182

**Published:** 2015-04-28

**Authors:** Lubna S. Abdalrahman, Harrington Wells, Mohamed K. Fakhr

**Affiliations:** Department of Biological Science, The University of Tulsa, Tulsa, OK 74104, USA; E-Mails: Lubna-abdalrahman@utulsa.edu (L.A.); Harrington-wells@utulsa.edu (H.W.)

**Keywords:** *Staphylococcus aureus*, antibiotic resistance, toxins, toxin genes, prevalence, beef livers, retail beef, retail pork, foodborne pathogens, retail meat

## Abstract

*Staphylococcus aureus* is one of the top five pathogens contributing to acquired foodborne illnesses causing an estimated quarter million cases every year in the US. The objectives of this study were to determine the prevalence of Methicillin Susceptible *S. aureus* (MSSA) and Methicillin Resistant *S. aureus* (MRSA) in retail beef livers, beef, and pork meats sold in Tulsa, Oklahoma and to characterize the recovered strains for their virulence and antimicrobial resistance. Ninety six chilled retail beef (50 beef livers and 46 beef other cuts), and 99 pork meat samples were collected. The prevalence in beef livers was 40/50 (80%) followed by other beef cuts 23/46 (50%) then pork 43/99 (43.3%). No isolates were positive for MRSA since none harbored the *mecA* or *mecC* gene. A total of 334 recovered *S. aureus* isolates (143 beef livers, 76 beef, and 115 pork isolates) were screened for their antimicrobial susceptibility against 16 different antimicrobials and their possession of 18 different toxin genes. Multidrug resistance was more prevalent in the pork isolates followed by beef then beef livers. The prevalence of enterotoxin genes such as *seg*, *seh*, and *sei* and the toxic shock syndrome gene *tst* was higher in the pork isolates than in the beef ones. The hemolysin genes, particularly *hlb*, were more prevalent in isolates from beef livers. Molecular typing of a subset of the recovered isolates showed that they are highly diverse where *spa* typing was more discriminatory than PFGE. The alarmingly high incidence of *S. aureus* in retail beef livers in this study should raise awareness about the food safety of such meat products.

## 1. Introduction

Foodstuff contamination with *Staphylococcus aureus* such as in dairy products and retail meats may occur as a result of poor hygiene during handling the food or directly from infected food-producing animals [1]. Symptoms of *Staphylococcus* poisoning which include diarrhea, abdominal cramps, vomiting, and nausea occur within 24 h after consuming toxin-contaminated food [2]. Most clinical signs of staphylococcal food poisoning are self-limited and disappear within 24–48 h after ingestion of the toxins. Death is rare and usually occurs in infants or the elderly [3]. More recent reviews are available discussing *S. aureus* in food and livestock [4,5,6].

Staphylococcal food poisoning has been considered an important cause of food-borne diseases around the world [7]. Some studies reported that Staphylococcal food poisoning has been the third most common cause of food-borne diseases in the last few decades worldwide [8]. Food-borne outbreaks in Europe between 1993 and 1998 were about 5.1% *S. aureus* [9]. *Staphylococcus aureus* is listed among the top five pathogens contributing to domestically acquired foodborne illnesses causing as many as 241,148 cases annually in the United States [10]. Both *Staphylococcus aureus* and MRSA have been isolated from chicken, pork, beef and others. For instance, one study in Japan found two isolates of MRSA out of 444 samples of raw chicken meats at 145 different supermarkets [11]. Other surveys in the Netherlands and Canada reported 20–40% of samples from pigs were positive for MRSA [12,13]. New different clones of MRSA that were related to pigs and cattle farming were detected in the Netherlands [14,15]. Recently, few studies reported variable prevalence of *S. aureus* and/or MRSA in retail meats at different US locations including Louisiana [16], Maryland, USA [17], Detroit, Michigan [18], Iowa [19], Minnesota, and New Jersey [20], Georgia [21], and North Dakota [22,23].

*S. aureus* is a bacterium of major concern worldwide due to its resistance to different types of antibiotics including beta-lactam antimicrobials [24]. A study from Louisiana [25] found that most *S. aureus* in different types of meat were commonly resistant to penicillin (71%), ampicillin (68%), tetracycline (67%), erythromycin (30%), clindamycin (18%), oxacillin with up to 2% of sodium chloride (14%), levofloxacin (13%), ciprofloxacin (13%), gentamicin (3%), dapfopristtin (3%), chloramphenical (2%), and moxifloxacin (1%). Another research study in Italy found that most *S. aureus* strains isolated from different foods were resistant to several types of antibiotics [1].

*S. aureus is* considered as one of the most dangerous pathogenic bacteria due to the production of a variety of extracellular protein toxins such as toxic shock syndrome toxin 1 (TSST-1), exfoliative toxin (ET), coagulase, hemolysins, and at least 15 types of enterotoxins [26]. Toxic shock syndrome of *S. aureus* causes staphylococcal scarlet fever. The production of exfoliative toxin of this bacterium causes staphylococcal scalded-skin syndrome, also known as Ritter’s disease [27]. Toxic shock syndrome toxin-1 (TSST-1) and enterotoxins (ETA, ETB) are the main ones responsible for toxin syndromes and food-borne diseases in humans and animals. Both of them belong to a family of superantigens [28]. Most *S. aureus* isolates produce different types of enterotoxins that are considered the main cause of food poisoning [27]. Many research studies have detected the prevalence of different types of toxin genes that are produced by *S. aureus* in different types of meats. For example, one of the studies by Pu *et al.* (2011) concluded that the high percentage of staphylococcal enterotoxins from Louisiana retail meat was 66% for *seg* and *sei*, 20% for *seh*, 15% for *sed*, 13% for *sej*, and 1% for *sea* [25]. Another research study in Korea reported different types of toxin genes in raw meats, which included toxic shock syndrome-1 (TSST-1) and enterotoxins [29].

Only a limited number of studies investigating the prevalence of MRSA in the food chain in the US were conducted in the last few years [17]. The number of studies discussing the prevalence of *S. aureus* in beef livers is very limited in the literature [30]. The objectives of this study were to determine the prevalence of *Staphylococcus aureus* and MRSA in retail beef livers, beef, and pork meats sold in the Tulsa, Oklahoma area and to characterize the recovered strains for their virulence and antimicrobial resistance.

## 2. Results and Discussion

### 2.1. Prevalence of Staphylococcus aureus and MRSA in Beef Livers, Beef and Pork

A total of 195 chilled retail beef and pork meat samples were purchased from several Tulsa area grocery stores. The samples were purchased weekly from January to June of 2010. There were 96 beef samples and 99 pork samples used in this study (Table 1). Fifty of the beef samples were beef livers and 46 were from other beef cuts like steak, shoulder, stew, neck, and bone in, *etc.* (Table 1). As shown in Table 1, the overall prevalence of *S. aureus* in beef was 63/96 (65.6%), while 43/99 (43.3%) of pork samples were contaminated with *S. aureus*. Also the prevalence of *Staphylococcus aureus* in beef livers 40/50 (80%) was higher than for other beef cuts (50%) (Table 1). So, the prevalence of *S. aureus* was higher in beef livers (80%) followed by beef (50%) then pork (43%). No isolates of beef livers, beef, or pork samples were positive for MRSA since none of them carried *mecA* or *mecC* genes.

**Table 1:** Prevalence of *Staphylococcus aureus* in the beef livers, beef, and pork samples.

Prevalence of *Staphylococcus aureus*			
Beef			Pork np/n (%)
Beef Liver *np/n (%)	Beef (Other Cuts) np/n (%)	Total np/n (%)	
40/50 (80%)	23/46 (50%)	63/96 (65.6%)	43/99 (43.3%)

* np: Number of positive samples; n: Number of samples collected.

Statistical comparison of beef livers, beef cuts and pork showed that significant differences exist among the three groups in incidence of *S. aureus* (*X*^2^ = 18.36, df = 2, *p* < 0.001). Thus, we went on to make pairwise comparisons. Beef cuts and pork where not significantly different (*X*^2^ = 0.55, df = 1, *p* > 0.25), and so these two groups were combined for comparison to beef livers. Beef livers when compared to {beef cuts + pork} were significant different in the incidence of *S. aureus* (*X*^2^ = 17.82, df = 2, *p* < 0.001). Beef livers harbor *S. aureus* more often than cuts of beef or pork.

Also, the overall prevalence of *S. aureus* in beef in this study was (65.6%) (Table 1), which is significantly higher than a study that showed 20% (comparison of our data to a model with 20% incidence: *X*^2^ = 124.90, df = 1, *p* < 0.001) [16], and one that showed 37% (comparison of our data to a model with 37% incidence: *X*^2^ = 33.75, df = 1, *p* < 0.001) [31] of beef samples were contaminated with *S. aureus.* Another research study in the Netherlands reported that the prevalence of *S. aureus* in beef was 33.3% [32], which is also significantly different from our finding. We clearly see that our study represents the highest prevalence of *S. aureus* in beef, but we attribute this to the fact that we included samples from beef livers that showed very high prevalence of 80%. The percentage of *S. aureus* in pork in our study was 43.3% (Table 1), which is almost in agreement with other published studies [31,32]. In Louisiana, a study found 45.6% of pork samples contained *S. aureus*, as did 20% of beef samples, while MRSA was found in 5.6% of pork samples and 3.3% of beef samples [16]. *S. aureus* was isolated from 56% of ground turkey, 28% of ground beef and only 12% of ground pork samples collected from Maryland, USA [17]. *S. aureus* was found in 20.5% of retail beef in Detroit, Michigan where two isolates were MRSA [18]. In another study in Iowa, 18.2% of retail pork and 6.9% of retail beef was positive for *S. aureus* where two of the pork isolates were identified as MRSA [19]. *S. aureus* was isolated from 64.8% of retail pork products in Iowa, Minnesota, and New Jersey in 2012 where 6.6% of the isolated strains were MRSA [20]. In a more recent study in Georgia, USA, *S. aureus* was isolates from 45% of retail pork and 63% of beef products where 3% of beef and 4% of pork were contaminated with MRSA [21]. Another study in North Dakota, USA showed a *S. aureus* prevalence of 49.3% in retail pork where 7% were MRSA [22]. A later study showed the prevalence of *S. aureus* in retail beef in Fargo, ND to be 9/36 and in retail pork to be 25/37 with slightly higher detection when realtime PCR was used [23]. An older study showed the prevalence of staphylococci to be 42% in beef liver and 27% in pork chops [30]. There are many factors that make the comparisons between different studies difficult and also these factors may contribute to the variety of positive percentages. Factors like different processing facilities, sampling, geographic locations, brand, isolation methods, and collection time can explain the variable prevalence of *S. aureus* among different studies in the literature.

### 2.2. Antimicrobial Resistance Screening

A total of 334 *S. aureus* recovered isolates (219 beef isolates [143 beef livers and 76 other beef cuts] and 115 pork isolates) were subjected to antimicrobial resistance profiling against 16 different antimicrobials that belong to ten different antibiotic classes (Table 4). As shown in Table 2 the percentage of resistance of the pork isolates was higher than beef isolates for all the antibiotics tested. The percentage of resistance of other beef cuts isolates was also higher than in beef liver isolates for almost all the antibiotics tested except rifampin and vancomycin. Resistance to oxacillin and cefoxitin was higher in the pork isolates (>40%) than in the beef ones despite the fact that no isolates in this study contained the *mecA* or *mecC* gene, so no genotypic MRSA was detected.

**Figure 1:**
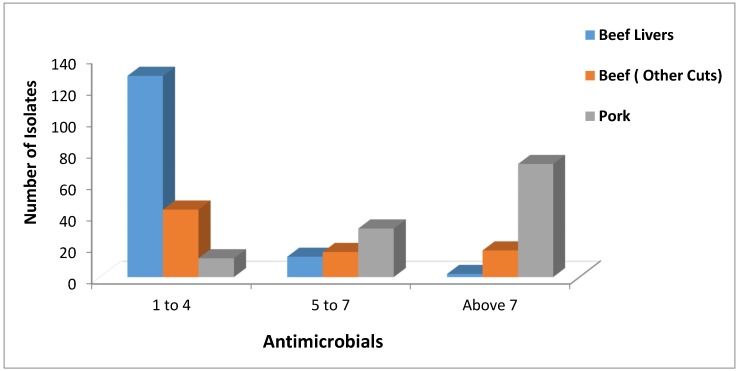
Distribution of the total number of *Staphylococcus aureus* isolates isolated from beef livers, beef, and pork according to their Multidrug Resistance (MDR) to one to four antimicrobials, five to seven antimicrobials, and more than seven antimicrobials of the 16 antimicrobials tested.

The distribution of Multidrug Resistance (MDR) among the 219 *S. aureus* beef isolates (143 beef livers and 76 other beef cuts) was as follows: 128 beef liver isolates and 43 other beef cuts isolates were resistant to one to four antimicrobials, 13 beef liver isolates and 16 other beef cuts isolates were resistant to five to seven antimicrobials, and two beef liver isolates and 17 other beef cuts isolates were resistant to more than seven antimicrobials (Figure 1). Also, the distribution of Multidrug Resistance (MDR) among the 115 *S. aureus* pork isolates was as follows: 12 isolates resistant to one to four antimicrobials, 31 isolates resistant to five to seven antimicrobials, and 72 isolates resistant to more than seven antimicrobials (Figure 1). Most of the pork isolates were highly multidrug resistant being resistant to more than seven antimicrobials. Also, most beef livers isolates were resistant to one to four antimicrobials indicating a lower level of multidrug resistance than the beef and pork ones.

**Table 2:** Antimicrobial resistance of the *Staphylococcus aureus* beef livers, beef, and pork isolates against 16 different antimicrobials. A test of the three *S. aureus* populations (beef livers, beef cuts, pork) for homogeneity in distribution of positive isolates across populations was performed for each antibiotic. Results: ** for *p* < 0.01, * for *p* < 0.05, and ^ns^ for not significant.

Antimicrobial Resistance				
Antibiotic	Beef			Pork np/n (%)
	Beef Livers * np/n (%)	Beef (Other Cuts) np/n (%)	Total np/n (%)	
azithromycin **	5/143 (3.5)	31/76 (40.8)	36/219 (16.4)	75/115 (65.2)
ciprofloxacin **	1/143 (0.7)	15/76 (19.7)	16/219 (7.3)	47/115 (40.9)
gentamicin **	8/143 (5.6)	22/76 (28.9)	30/219 (13.7)	68/115 (59.1)
oxacillin **	10/143 (6.9)	6/76 (7.9)	16/219 (7.3)	56/115 (48.7)
cefoxitin **	11/143 (7.7)	8/76 (10.5)	19/219 (8.7)	47/115 (40.9)
tetracycline **	21/143 (14.7)	33/76 (43.4)	54/219 (24.7)	109/115 (94.8)
vancomycin **	12/143 (8.4)	6/76 (7.9)	18/219 (8.2)	49/115 (42.6)
doxycycline **	10/143 (6.9)	26/76 (34.2)	36/219 (16.4)	96/115 (83.5)
trimethoprirm/ sulfamethazole **	1/143 (0.7)	2/76 (2.6)	3/219 (1.4)	22/115 (19.1)
clindamycin **	1/143 (0.7)	9/76 (11.8)	10/219 (4.5)	25/115 (21.7)
penicillin **	31/143 (21.7)	48/76 (63.2)	79/219 (36.1)	102/115 (88.7)
ampicillin ^ns^	142/143 (99.3)	76/76 (100)	218/219 (99.5)	115/115 (100)
kanamycin **	4/143 (2.8)	23/76 (30.3)	27/219 (12.3)	62/115 (53.9)
erythromycin **	8/143 (5.6)	31/76 (40.8)	39/219 (17.8)	63/115 (54.8)
rifampin *	21/143 (14.7)	6/76 (7.9)	27/219 (12.3)	25/115 (21.7)
chloramphenicol **	3/143 (2.1)	6/76 (7.9)	9/219 (4.1)	35/115 (30.4)

np: Number of positive isolates; n: Number of isolates collected.

A test of the three *S. aureus* populations (beef livers, beef cuts, pork) for homogeneity in distribution of MDR (three categories: 1–4, 5–7, 8+) showed that there were significant difference (*X*^2^ = 173.16, df = 4, *p* < 0.001). Thus, we went on to make pairwise comparisons. All three pairwise comparisons were significant (*X*^2^ ≥ 37.41, df = 2, *p* < 0.001 in each case). Beef livers showed the lowest and pork the greatest MDR levels.

In this study, beef isolates were highly resistant to ampicillin (36.1%) followed by tetracycline (24.7%) (Table 2). High resistance in pork isolates in our study were as follows: Ampicillin (100%), tetracycline (94.8%), penicillin (88.7%), doxycycline (83.5%); however, in a previous study from Louisiana, antimicrobial resistance of beef and pork samples was slightly lower: Penicillin (71%), ampicillin (68%) and tetracycline (67%) [16]. In a different study, 70% and 73% of *S. aureus* isolated strains were resistant to ampicillin and penicillin, respectively [33]. In another study, more turkey and pork isolates were resistant to ampicillin, penicillin and tetracycline than the beef ones [17]. Resistance to vancomycin was also significantly higher in pork isolates compared to beef isolates (Table 2). In a recent study, the authors indicated that high levels of in-feed zinc and other disinfectants used in commercial swine herds might be possible drivers in the selection and persistence of MRSA in the herds [33]. They also suggested that these agents may be co-selecting for other antimicrobial resistance genes [33]. It is reasonable to propose that the higher vancomycin resistance reported in pork compared to beef in our study might be resulting from the heavy use of antimicrobial feed additives, heavy metals and disinfectants in swine production. In the present study, MDR was common among *S. aureus* isolates. Isolates that were resistant to more than seven antimicrobials were prevalent in the pork isolates. Another study reported that isolates resistant to three or more antimicrobials were found in turkey, followed by those from pork, and chicken [31].

In our study, there was a significant number of *S. aureus* strains (>40% in the pork isolates) that showed the same phenotypic behavior as MRSA by showing resistance to both cefoxitin and oxacillin (Table 2). Genotypically, they were not MRSA, since they did not possess the *mecA* or the *mecC* gene. Phenotypic MRSA isolates that do not contain the *mecA* gene were also detected previously [17]. In a study in Portugal, 38% of *S. aureus* isolates from various foods were resistant to oxacillin but only 0.68% showed the presence of *mecA* gene [34]. This behavior can be due to the overproduction of Beta-lactamase enzymes or might be due to the presence of a MRSA variant *mecA* gene that cannot be detected with the currently available PCR primers. Two recent studies exploring the presence of such variant *mec*A genes support this hypothesis [35,36].

### 2.3. Toxin Gene Possession Screening

A total of 334 *Staphylococcus aureus* isolates (219 beef isolates (143 beef livers and 76 other beef cuts) and 115 pork isolates) were screened for 18 different toxin genes that belong to six different toxin gene groups (Table 3). As shown in Table 3, *S. aureus* hemolysin genes were found at a higher percentage in beef and pork than other groups of toxin genes. Also, no isolates of beef and pork harbored enterotoxin genes *seb-sec* or *see*, the exfloliative toxin genes *eta* or *etb*, or the Leucocidin gene *lukM*. Also, the prevalence of hemolysin genes *hla* (93.2%) and *hld* (93.2%) in beef isolates were higher than in the pork ones, where *hla* was 86.9% and *hld* was 86.9%. Hemolysin gene *hlb* was present more often in beef than in pork. No isolates out of 219 of *S. aureus* from beef was positive for the entoretoxin gene *sec*, while one isolate (0.9%) from pork was positive for this gene. Also, two isolates out of 219 of *S. aureus* from beef were positive for the entoretoxin genes *sed* (0.9%) and *sej* (0.9%), while no isolates from pork was positive for these two genes. The percentage of toxic shock syndrome toxin 1 gene *tst* from pork isolates (13%) was higher than beef isolates (1.8%). One isolate out of 115 of *S. aureus* from pork was positive for the PVL gene *lukS-lukF* (0.9%), while no isolates from beef was positive for this particular gene. As shown in Table 3, the percentage of the *lukE*-*luk*D gene in other beef cuts isolates was higher than from beef liver isolates. Also, the percentage of the *hlb* gene in beef liver isolates was higher than from other beef cuts isolates.

**Table 3:** Toxin gene screening of the *Staphylococcus aureus* beef livers, beef, and pork isolates to 18 different toxin genes. A test of the three *S. aureus* populations (beef livers, beef cuts, pork) for homogeneity in distribution of positive isolates across populations was performed for each toxin gene. Since many expected cells are small Yates correction was used throughout. Results: *** for *p* < 0.001, ** for *p* < 0.01, * for *p* < 0.05, and ^ns^ for not significant.

Prevalence of Toxin Genes				
Toxin Gene	Beef			Pork np/n (%)
	Beef Livers * np/n (%)	Beef ( Other Cuts) np/n (%)	Total np/n (%)	
*sea ^ns^*	0/143 (0.0)	2/76 (2.6)	2/219 (0.9)	0/115 (0)
*seb-sec ^ns^*	0/143 (0.0)	0/76 (0.0)	0/219 (0)	0/115 (0)
*sec ^ns^*	0/143 (0.0)	0/76 (0.0)	0/219 (0)	1/115 (0.9)
*sed ^ns^*	0/143 (0.0)	2/76 (2.6)	2/219 (0.9)	0/115 (0)
*see ^ns^*	0/143 (0.0)	0/76 (0.0)	0/219 (0)	0/115 (0)
*seg* **	4/143 (2.8)	5/76 (6.6)	9/219 (4.1)	18/115 (15.7)
*seh ^ns^*	0/143 (0.0)	0/76 (0.0)	0/219 (0)	36/115 (31.3)
*sei* **	2/143 (1.4)	11/76 (14.5)	13/219 (5.9)	8/115 (6.9)
*sej ^ns^*	1/143 (0.7)	1/76 (1.32)	2/219 (0.9)	0/115 (0)
*tst* ***	4/143 (2.8)	0/76 (0.0)	4/219 (1.8)	15/115 (13.0)
*eta ^ns^*	0/143 (0.0)	0/76 (0.0)	0/219 (0)	0/115 (0)
*etb ^ns^*	0/143 (0.0)	0/76 (0.0)	0/219 (0)	0/115 (0)
*lukE-lukD* ***	12/143 (8.4)	24/76 (31.6)	36/219 (16.4)	41/115 (35.7)
*lukM ^ns^*	0/143 (0.0)	0/76 (0.0)	0/219 (0)	0/115 (0)
*hla* ***	141/143 (98.6)	63/76 (82.9)	204/219 (93.2)	100/115 (86.9)
*hlb* ***	128/143 (89.5)	38/76 (50.0)	166/219 (75.8)	41/115 (35.7)
*hld* ***	141/143 (98.6)	63/76 (82.9)	204/219 (93.2)	100/115 (86.9)
*lukS-lukF ^ns^*	0/143 (0.0)	0/76 (0.0)	0/219 (0)	1/115 (0.9)

* np: Number of positive isolates; n: Number of isolates collected.

In the Louisiana study, most of their beef and pork isolates were positive for *seg, sei* (66%), followed by *seh* (20%), *sed* (15%), *sej* (13%), and *sea* (1%) and no isolates were positive for enterotoxins *sec*, *seb*, or *see*, the toxic shock syndrome toxin 1 gene *tst*, or the exfloliative toxin genes *eta*, or *etb* [16]. The presence of *tst* in 13% of our pork isolates is alarming. The higher prevalence of hemolysin genes, particularly *hlb*, in beef livers might be due to the availability of blood in the liver. Other than the Louisiana study mentioned above, the rest of the US studies concerning the prevalence of *S. aureus* and MRSA in retail meats did not screen their recovered isolates for the possession of several toxin genes. A study in Italy reported that the prevalence of enterotoxin genes for *S. aureus* was 58.8% in meat and dairy products [1]. Therefore, as was the case in the prevalence and antimicrobial resistance data, toxin gene possession can also vary by meat type, processing facility, location, and brand. It is interesting to report that the prevalence of toxin genes in retail meat samples in this study appears generally lower than those previously reported in human *Staphylococcus aureus* (both MSSA and MRSA) with the exception of hemolysin genes where the prevalence was only slightly lower than in the human strains [37].

The high prevalence of *S. aureus* in retail meats in our study, particularly in beef livers (80%), is of concern, particularly that a recent study proved the potential of MRSA transfer from retail pork onto food contact surfaces and the possibility of consumer exposure [38]. Another study demonstrated that MRSA acquired on pig farms can be transferred through processing in the slaughter house [39]. Colonization of pork butchers with livestock associated MRSA (LA-MRSA) that was suggested to be acquired by cross-contamination from pork was recently proposed [40].

The high prevalence of *S. aureus* in retail beef livers is alarming and might be due to cross contamination since livers are recovered from several cows in slaughter houses and possibly piled up together. In *Campylobacter*, Ghafir *et al.* [41] suggested that the high level of recovery of the bacterium from livers is probably due to the fact that the liver surface stays moist, which might protect this foodborne pathogen. A possible explanation can be also true in *S. aureus*. Human handlers are possible sources of contaminating beef livers in slaughter houses. The risk of the high prevalence of *S. aureus* in beef liver in our study could be more severe due to the fact that livers are usually lightly cooked to avoid the undesired taste of overcooking. Adding to this risk is the fact that some *S. aureus* enterotoxins are heat stable. We have recently also found high prevalence of *Campylobacter* in beef livers [42] and chicken livers [43].

The high prevalence of *S. aureus* strains on retail beef livers, beef, and pork sold in Oklahoma that belong to major brands is alarming. While the absence of MRSA in our collected retail meats is considered good news, a good percentage of the isolated non-MRSA *Staphylococcus aureus* strains were highly resistant to multiple antimicrobials and possess several toxin genes. More prevalence studies should be conducted in the US to gather more data that can help in reducing the presence of this important foodborne pathogen in retail meats.

### 2.4. Molecular Typing Using Spa Typing and PFGE

**Figure 2:**
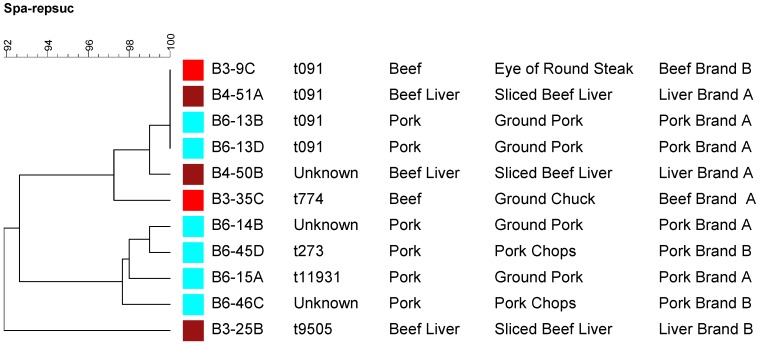
A dendrogram showing *spa* typing for a subset of the recovered *Staphylococcus aureus* strains representing different meat sources, cuts, and brands. Strains isolated from the same meat source are labeled by the same color square.

**Figure 3:**
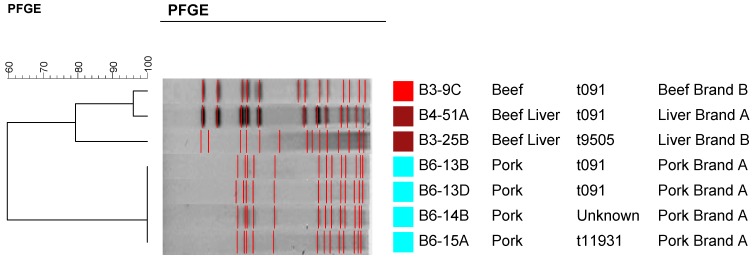
Pulsed Field Gel Electrophoresis (PFGE) patterns of a subset of the recovered *Staphylococcus aureus* strains representing different meat sources and brands showing their corresponding *spa* types. Four strains were not typable by PFGE and hence they are not shown in the dendrogram.

A subset of *S. aureus* recovered strains (three from beef livers, two from beef other cuts, and six from pork) and representing different brands and cuts were subjected to molecular typing by spa typing (Figure 2) and Pulsed Field Gel Electrophoresis (PFGE) (Figure 3). As it is shown in Figure 2, there was no direct correlation between a specific *spa* type and a particular meat source except that four of the pork isolates were clustered together. Strains isolated from each of the meat sources showed high level of diversity in regards to their spa types (Figure 2). Even though four strains were non-typable by PFGE, PFGE was able to separate the strains according to their meat source into two major clusters (Figure 3). As it is shown in Figure 3, pork isolates were clustered together in one cluster separate from those isolated from beef. It is worthy to note that strains of the same PFGE pattern showed different *spa* types (Figure 3). So, *spa* typing appears to be more discriminatory than PFGE in this regard which was previously reported [37]. It is also clear that *S. aureus* strains isolated from beef and pork in this study were highly diverse using *spa* typing (Figure 2). Nasal carriage and clinical MSSA isolates were previously reported to be more variable than CA-MRSA [37].

## 3. Experimental Section

### 3.1. Isolation of *Staphylococcus aureus* from Retail Meat Samples

Meat samples were collected from several different grocery stores in the Tulsa, Oklahoma area on weekly bases from January to June 2010. A total of 195 chilled retail beef and pork meat samples were used in this study (96 beef samples [50 beef livers and 46 beef other cuts] and 99 pork samples) (Table 1). Meat samples were purchased from nine grocery stores that belong to six different franchises chains at variable locations in the city. The collected beef samples belonged to nine different brands while the pork ones were from seven brands. Pork and beef samples other than livers were from different cuts such as, steak, stew, shoulder, neck, and bone *etc.* Samples were selected to be variable as possible with different expiration dates and production codes. Meat samples were rinsed with 10 mL of buffered peptone water (BPW) (BPW; EMD, Gibbstown, NJ, USA) in sterile plastic bags (VWR Scientific, Radnor, PA, USA) and then massaged by hand for 5 min. Then, 10 mL the rinse was enriched in 10 mL of enrichment broth of 2 X Trypticase Soy Broth with 10% sodium chloride and 1% sodium pyruvate. The enrichment was incubated at 37 °C for 24 h and then streaked to Baird Parker (BP) selective media plates. Later, all plates were incubated at 37 °C for 48 h [16]. Four suspected *S. aureus* colonies (those that have black colonies surrounded by 2–5 mm clear zones) were selected and streaked to Trypticase Soy Agar (TSA) plates and subcultured for confirmation on MSA (Mannitol Salt Agar) plates.

### 3.2. DNA Extraction

The single cell lysing buffer (SCLB) method was used to extract bacterial DNA for polymerase chain reaction (PCR) from prospective *S. aureus* cultures as described previously [44].

### 3.3. PCR Identification

A multiplex PCR reaction was used to identify the isolated suspected *S. aureus* by using specific primers for *S. aureus* and MRSA to amplify a 108 bp [45] and a 312 bp [12] fragments respectively. The multiplex PCR was performed as described previously [16]. Isolates showing resistance to cefoxitin and/or oxacillin were subjected to PCR confirmation using a second set of MRSA primers that amplify a 533 bp *mecA* fragment [46] and two other variant MRSA *mecA* primer sets (also known as *mecC*) that amplify 356 bp [35] and 1800 bp [36] fragments to confirm the MRSA phenotype.

### 3.5. Antimicrobial Resistance Screening

A total number of 334 *Staphylococcus aureus* isolates (219 beef isolates (143 beef livers and 76 other beef cuts) and 115 pork isolates) were subjected to antimicrobial susceptibility testing against 16 different antimicrobials that belong to ten different antibiotic classes (Table 4). Isolates were grown on Mueller-Hinton (MH) agar (Difco) and incubated for 48 h at 37 °C. Cultures were then added to Mueller-Hinton broth (Difco), adjusted to turbidity equal to a 0.5 McFarland standard, and inoculated onto 6-inch MH agar plates supplemented with the appropriate antimicrobial at different concentrations (Table 4) including the breakpoint established for each antimicrobial according to the Clinical and Laboratory Standards Institute (CLSI) when available [47]. The plates were incubated at 37 °C for 48 h. The plates were read for growth or no growth and denoted as resistant or susceptible, respectively, according to the breakpoints for each of the 16 tested antimicrobials (Table 4).

**Table 4:** A list of the 16 tested antimicrobials, their classes, the concentrations used for susceptibility testing, and the breakpoints used for each antimicrobial.

Antimicrobial Class	Antimicrobials	Conc. 1 (µg/mL)	Conc. 2 (µg/mL) (Break point)	Conc. 3 (µg/mL)	Conc. 4 (µg/mL)
β-Lactams	penicillin	0.125	0.25	0.5	1
	ampicillin	0.25	0.5	1	2
	oxacillin + 2% Nacl	2	4	8	16
	cefoxitin + 2% Nacl	4	8	16	32
Tetracyclines	tetracycline	8	16	32	64
	doxycycline	8	16	32	64
Macrolides	azithromycin	4	8	16	32
	erythromycin	4	8	16	32
Aminoglycosides	kanamycin	32	64	128	256
	gentamicin	8	16	32	64
Fluoroquinolones	ciprofloxacin	2	4	8	16
Lincosamides	clindamycin	2	4	8	16
Phenicols	chloramphenicol	16	32	64	128
Glycopeptides	vancomycin	16	32	64	128
Rifamycines	rifampin	2	4	8	16
Sulfonamides	trimethoprim/sulfamethoxazole	2/38	4/76	8/152	16/304

### 3.6. Prevalence of Toxin Genes

A total of 334 *Staphylococcus aureus* isolates (219 beef isolates (143 beef livers and 76 other beef cuts) and 115 pork isolates) were screened for 18 different toxin genes that belong to six different toxin gene groups. Multiplex PCR was used to detect 18 different toxin genes of *S. aureus* isolates that include enterotoxins (*sea*, *seb-sec*, *sec*, *sed*, *see*, *seg*, *seh*, *sei*, *sej*), toxic shock syndrome toxin 1 (*tst*), exfoliative toxins (*eta*, *atb*), leucocidins (*lukE-lukD*, *lukM*), Panton-Valentine leucocidin (PVL) (*lukS-lukF*), and hemolysins (*hla*, *hlb*, *hld*). Three multiplex reactions (A, B, and C), each of which included six toxin genes, were performed (Table 5). The multiplex PCRs targeting the toxin genes were performed in a 20 μL reaction solution that contained 10 μL of Green Master Mix (Promega), 2 μL of sterile water, 2 μL of the DNA template and 0.5 μL of each of the toxin gene primers. The thermocycling protocol for toxin genes included an initial denaturation at 95 °C for 5 min, followed by 30 cycles of denaturation (94 °C for 1 min), annealing (57 °C for 1 min), and extension (72 °C for 1 min), ending with an extension at 72 °C for 7 min. Then 10 μL of the multiplex PCR product was added to a 2% (wt/vol) agarose gel in 1x Tris-acetate-EDTA (TAE) buffer. A 100–10,000 bp ladder was used as a molecular marker. The electrophoresis was run at 100 mV for 1 h and 10 min. The gel was stained for 15 min using *ethidium bromide* and distained in distilled water for 30 min. The DNA bands were visualized and recorded under UV using a gel documentation system. The expected amplicon band sizes of *S. aureus* toxin genes are shown in Table 5. At least one PCR amplicon of each positively reported toxin gene was sequenced using the same amplifying primers to ensure the accuracy of the PCR amplification.

**Table 5:** Multiplex PCR primers, reaction sets, and references for toxin genes screening.

Toxin Gene	Size (bp)	Primer sequences (5'-3')	Multiplex PCR Reaction Set	Ref.
*sea*	521	GCA GGG AAC AGC TTT AGG C	A	[48]
		GTT CTG TAG AAG TAT GAA ACA CG		
*seb-sec*	665	ATG TAA TTT TGA TAT TCG CAG TG	A	[48]
		TGC AGG CAT CAT ATC ATA CCA		
*sec*	284	CTT GTA TGT ATG GAG GAA TAA CAA	A	[48]
		TGC AGG CAT CAT ATC ATA CCA		
*sed*	385	GTG GTG AAA TAG ATA GGA CTG C	A	[48]
		ATA TGA AGG TGC TCT GTG G		
*see*	171	TAC CAA TTA ACT TGT GGA TAG AC	A	[48]
		CTC TTT GCA CCT TAC CGC		
*seg*	328	CGT CTC CAC CTG TTG AAG G	A	[48]
		CCA AGT GAT TGT CTA TTG TCG		
*seh*	359	CAA CTG CTG ATT TAG CTC AG	B	[48]
		GTC GAA TGA GTA ATC TCT AGG		
*sei*	466	CAA CTC GAA TTT TCA ACA GGT AC	B	[48]
		CAG GCA GTC CAT CTC CTG		
*sej*	142	CAT CAG AAC TGT TGT TCC GCT AG	B	[48]
		CTG AAT TTT ACC ATC AAA GGT AC		
*tst*	560	GCT TGC GAC AAC TGC TAC AG	B	[48]
		TGG ATC CGT CAT TCA TTG TTA A		
*eta*	93	GCA GGT GTT GAT TTA GCA TT	B	[26]
		AGA TGT CCC TAT TTT TGC TG		
*etb*	226	ACA AGC AAA AGA ATA CAG CG	B	[26]
		GTT TTT GGC TGC TTC TCT TG		
*lukS-lukF*	433	ATC ATT AGG TAA AAT GTC TGG ACA TGA TCC A	C	[27]
		GCA TCA AST GTA TTG GAT AGC AAA AGC		
*lukE-lukD*	269	TGA AAA AGG TTC AAA GTT GAT ACG AG	C	[27]
		TGT ATT CGA TAG CAA AAG CAG TGC A		
*lukM*	780	TGG ATG TTA CCT ATG CAA CCT AC	C	[27]
		GTT CGT TTC CAT ATA ATG AAT CAC TAC		
*hla*	209	CTG ATT ACT ATC CAA GAA ATT CGA TTG	C	[27]
		CTT TCC AGC CTA CTT TTT TAT CAG T		
*hlb*	309	GTG CAC TTA CTG ACA ATA GTG C	C	[27]
		GTT GAT GAG TAG CTA CCT TCA GT		
*hld*	111	AAG AAT TTT TAT CTT AAT TAA GGA AGG AGT G	C	[27]
		TTA GTG AAT TTG TTC ACT GTG TCG A		

### 3.7. Molecular Typing of Staphylococcus aureus Isolates

A subset of the recovered *S. aureus* isolates were subjected to molecular typing using *spa* typing and Pulsed Field Gel Electrophoresis (PFGE). Isolates were chosen to represent a percentage of the positive samples of each meat source (beef, beef livers, and pork). Within each meat source, isolates were chosen to be as variable as possible to represent different brands, meat cuts, dates of collection, antimicrobial susceptibility and toxin profiles. The molecular typing using *spa* was done according to published primers and protocols [49] and *spa* types were assigned using the BioNumerics Softwatre (Applied Math, Austin, TX, USA). PFGE was performed according to the CDC protocol [50]. The digested plugs were run in Seakem agarose gel (Lonza, Allendale, NJ, USA) with 0.5× Tris-Borate EDTA (TBE) buffer (Amresco, Solon, OH, USA) to separate the bands on the CHEF Mapper PFGE system (Bio-Rad). Gel images were analyzed using BioNumerics software (Applied Maths, Austin, TX, USA). The banding patterns were clustered using Dice coefficients and unweighted pair group method, with arithmetic mean (UPGMA), and a 3% band tolerance.

## 4. Conclusions

The prevalence of *Staphylococcus aureus* in retail beef livers in this study was alarmingly high. The prevalence of this foodborne pathogen in retail beef and pork, while lower than beef livers, is still very significant. Multidrug resistance was generally higher in the pork isolates followed by the beef and beef liver ones. While no isolate harbored the *mecA* gene, a good percentage of the pork isolates were phenotypically similar to MRSA strains by being resistant to cefoxitin and oxacillin. Few of the *S. aureus* strains recovered from retail meats in this study possessed several toxin genes including enterotoxins. Molecular typing of a subset of the recovered isolates showed that they are highly diverse where *spa* typing was more discriminatory than PFGE. Even though the prevalence of enterotoxin genes in beef livers was lower than beef and pork meats, care should be taken not to leave beef livers for longer periods of time at room temperature prior to cooking to reduce the chance of thermostable enterotoxin production, particularly as livers are usually not overcooked.
